# Norwegian validation of the University Student Engagement Inventory

**DOI:** 10.3389/fpsyg.2026.1734409

**Published:** 2026-01-26

**Authors:** Trygve K. Løken, Natallia B. Hanssen, Kathrin Olsen, João Marôco

**Affiliations:** 1 Faculty of Education and Arts, Nord University, Bodø, Norway; 2 Intrepid Lab, ECEO, Universidade Lusófona & CETRAD, Centro de Estudos Transdisciplinares para o Desenvolvimento, UTAD, Lisbon, Portugal

**Keywords:** academic variables, dropout intention, psychometric analysis, student engagement, validation

## Abstract

This study reports on the validation of the University Student Engagement Inventory (USEI) in a Norwegian context. The USEI conceptualizes student engagement (SE) as a three-factor first-order construct encompassing cognitive, emotional, and behavioral dimensions and as a second-order construct. Psychometric analysis was conducted with a sample of 833 students from Norwegian universities and university colleges, representing both science, technology, engineering, and mathematics (STEM) and non-STEM fields (humanities and social sciences). Psychometric analysis is reported, including distributional properties, sources of evidence related to the internal structure, and external criteria. The paper presents an invariance analysis for area of study and gender. Criterion validity was evaluated with respect to students’ dropout intention, failed university courses, and use of medication to cope with study-related challenges. The USEI demonstrated good factorial construct validity and reliability for both the first- and second-order constructs. Higher levels of SE significantly predicted lower dropout intentions, fewer failed courses, and reduced medication use among Norwegian students. The study demonstrates that the USEI can deliver reliable, valid data on SE in the Norwegian context. It has predictive value for academic variables and is therefore important for policymakers and stakeholders in the education system.

## 1 Introduction

Entering higher education (HE) is a phase of adaptation for young individuals (Hernández-Torrano et al., 2020). Students must swiftly acclimate to new academic settings and expectations (Amdal and Hanssen, 2025; Hernández-Torrano et al., 2020), stricter time management, and elevated academic standards (Fredricks, 2015). They must also make independent decisions about their lives and studies while organizing their social and academic activities (Petronienė et al., 2024). Students’ success is often linked to their engagement (SE) in HE, and scholars across the globe have demonstrated that SE significantly influences students’ academic performance and learning (Balwant et al., 2019; Miller et al., 2021), mitigates the risk of burnout (Paloș et al., 2019; Sharif-Nia et al., 2023), and influences emotional intelligence and overall satisfaction with university life (Zhoc et al., 2019). Notably, highly engaged students tend to have fewer depressive symptoms and perform better academically than those who are disengaged (Esposito et al., 2022). Conversely, low SE can result in poor academic performance, burnout, reduced resilience, dissatisfaction, and higher dropout rates in HE (Calcatin et al., 2022; Christenson and Reschly, 2010; Marôco et al., 2016, 2020).

Concerning the measurement of SE in HE, various self-report measures methods are used, including questionnaires, behavioral analysis, interviews and behavioral observations (Fredricks and McColskey, 2012), as well as instruments assessing the cognitive, affective, and behavioral dimensions of SE (Buntins et al., 2021). For example, among the recent scales, the Higher Education Student Engagement Scale (HESES; Zhoc et al., 2019), the Australasian Survey of Student Engagement (Coates, 2008), the Utrecht Work Engagement Scale – Student version (UWES-SS) (Schaufeli et al., 2003), Beginning College Survey of Student Engagement [Beginning College Survey of Student Engagement (BCSSE), 2013], Study Process Questionnaire (R-SPQ-2F) (Biggs et al., 2001), Classroom Community Scale (Rovai, 2002), and National Survey of Student Engagement (NSSE) by Kuh (2003) are known.

However, many measurement instruments have faced several criticisms, including issues with construct definitions, dimensionality, and applicability to university students (Marôco et al., 2016), as well as the lack of information on how they measure SE (Buntins et al., 2021) and the lack of reporting reliability scores (Buntins et al., 2021).

In the search for a valid and reliable instrument that comprehensively assesses SE in HE, Marôco et al. (2016) developed the University Student Engagement Inventory (USEI) as a measuring instrument for SE in the HE context. This instrument has been found to be a psychometrically suitable instrument for assessing SE in HE (Laranjeira and Teixeira, 2025). Drawing on Fredricks’s conceptualization (2015), the USEI is based on a three-dimensional model of engagement that encompasses behavioral, emotional, and cognitive dimensions and SE as a second-order construct reflected along these three dimensions. Across 11 countries in Europe, Africa, the Americas, and Asia, studies examining the USEI have shown that it provides reliable and valid measures of university SE (Assunção et al., 2020; Freiberg-Hoffmann et al., 2022; Marôco et al., 2016; Sharif-Nia et al., 2023, 2024; Sinval et al., 2021). These findings support the USEI as a robust tool for understanding SE in HE across diverse cultural contexts. The USEI demonstrates satisfactory item sensitivity, factor validity for both the three-factor and second-order models, discriminant and convergent validity, and reliability across the three dimensions. Additionally, it exhibits strong measurement invariance across fields of study and gender and serves as a significant predictor of students’ dropout intentions, course completion rates, academic performance, and levels of burnout. These results strongly indicate that the USEI provides satisfactory evidence of validity for its internal structure, with its dimensions showing meaningful associations with key aspects of the university experience (Assunção et al., 2020; Marôco et al., 2020; Sharif-Nia et al., 2023).

Norway is not exempt from the global challenges of low SE in HE and its academic and social consequences. In Norway, approximately 25% of students in HE are estimated to discontinue their studies before completion (Andresen and Lervåg, 2022). Furthermore, mental health issues (Grøsland et al., 2022), prevalence of stress, anxiety, depression, NSSH (non-suicidal self-harm) and suicidality among students in HE (Knapstad et al., 2021; Sivertsen et al., 2025) are major concerns prompting HE institutions, researchers, and decision-makers to address these challenges.

Research on SE in HE in Norway remains scarce. Lycke and Handal’s (2016) review of Norwegian studies on SE identified seven relevant works. Four studies focus on engagement in nursing education (Alvsvåg and Førland, 2007; Christiansen, 2007; Lervåg, 1998; Løviknes, 2011). Berg and Erichsen (2014) explore engagement in relation to learning among students in management and leadership programs, while Bjuland and Mosvold (2014) examine student teachers’ engagement in relation to the development of learning communities. Langøy (2015) investigates HE students’ political engagement, defined as democratic participation in program development and the social dimensions of student life. More recent studies focus on the COVID-19 pandemic’s impact on students’ academic environments and their social and private lives (Petronienė et al., 2024), as well as on supportive study climate (Slåtten et al., 2021).

The ‘Study Barometer’ is an annual survey of Norwegian students in their second and fifth year of higher education, focusing on student satisfaction and quality in education. While it provides some insight into the behavioral dimension of student engagement and related factors, its indicators are dispersed across multiple indices with diverse measures that do not consistently align with a single overarching concept. Consequently, it cannot serve as a comprehensive index for assessing student engagement (Munthe, 2020).

In Norwegian research literature, SE is often described imprecisely in studies with other research aims and typically examined through small-scale qualitative investigations (Lycke and Handal, 2016). This underscores the need for larger-scale studies that employ a clear definition of the concept, aligned with established international frameworks and supported by explicit theoretical grounding (Lycke and Handal, 2016; Munthe, 2020). Although several SE scales have shown good psychometric properties in HE internationally, socioeconomic, linguistic and value differences may influence the scales’ validity in a specific cultural context. To the best of our knowledge, no nationally representative study has employed psychometrically robust tools to assess SE measurement across the Norwegian HE population. This gap is compounded by the absence of a nationally validated measure of SE in HE, limiting institutions’ capacity to identify at-risk students and implement preventive interventions. Given the paramount importance of SE in HE and the absence of a validated Norwegian version of the USEI for HE students, it is essential to carry out a study to fill this gap. This will result in a culturally appropriate tool to evaluate SE and guide educational practices within the Norwegian HE context.

This article aims to provide a Norwegian translation of the USEI and to examine its psychometric properties—including construct validity, discriminant and convergent validities, and reliability—its invariance by area of study and gender, and its criterion validity related to students’ academic coping (dropout intentions, failed university courses, and medication use for coping with study-related challenges) among Norwegian university students. Specifically, it sought to answer the following questions:

1. To what extent is the USEI valid within the Norwegian context?
2. To what extent is the USEI reliable within the Norwegian context?

We anticipate finding adequate psychometric results in the Norwegian sample and that SE is predictive of academic coping, consistent with previous research on the USEI conducted in other countries. In this study, the scale of interest for examining SE is the institutional level within HE, encompassing students across Norwegian HE institutions regardless of discipline or course.

## 2 Theoretical framework

The concept of engagement has been extensively examined in diverse contexts, including psychotherapeutic environments (Holdsworth et al., 2014), professional and organizational settings (Bakker et al., 2008), schools (Fredricks et al., 2004), and HE institutions (Hu and Kuh, 2002). Over the years, numerous definitions, conceptualizations, and models of engagement have been developed (for reviews, see Alrashidi et al., 2016; Fredricks et al., 2004; Sharif-Nia et al., 2023).

There are various definitions of SE in the literature. For example, Kuh and Hu (2001) define SE as students’ devotion to educationally purposeful activities, whereas Axelson and Flick (2010) describe it as students’ involvement or interest in their learning combined with their connection to their institutions, their classes, and one another. Esposito et al. (2022, p. 35) point out that the concept of engagement refers to an individual’s active participation and connection in social and institutional settings and serves as a key construct for social processes regarding innovation and development.

Scholars have identified multiple frameworks for examining SE in HE, each emphasizing different dimensions of how students connect with their learning environments. Esposito et al. (2022) and Kahu (2013) categorize four primary views of engagement in HE: (a) behavioral, focusing on students’ actions, participation, and effort in academic and co-curricular activities (Coates, 2008; Kahu, 2013; Kuh, 2009); (b) psychological, emphasizing intra-individual elements such as motivation, self-efficacy, and emotional investment (Fredricks et al., 2004; Kahu, 2013); (c) socio-cultural, which situates engagement within broader social and institutional contexts (Kahu, 2013; Mann, 2001); and (d) holistic, integrating these perspectives to account for the full complexity of the student experience (Bryson et al., 2009; Kahu, 2013).

This typology aligns with earlier conceptualizations such as Kahu’s (2013) influential model, which distinguishes behavioral, psychological, and socio-cultural dimensions while proposing a holistic synthesis. Similarly, Zepke (2015) argues that engagement research must move beyond a narrow behavioral focus to embrace social and cultural ecosystems, while Pickford (2015) presents a multidimensional model incorporating academic, emotional, and transactional aspects of engagement. More recent frameworks continue this integrative approach; examples include Reifenrath et al.’s (2024) operationalization of an integrated behavioral–psychological–psychosocial model and Castro’s (2024) bibliometric mapping of engagement research into behavioral, psychological, and multidimensional schools of thought. Collectively, these perspectives illustrate an ongoing effort to conceptualize SE as a multi-layered construct encompassing individual, social, and institutional dimensions. Nonetheless, this broad perspective has been criticized for neglecting SE’s emotional dimensions, particularly the relational connections students develop in an HE environment (Esposito et al., 2022). Fredricks et al. (2004) proposed a psychological perspective aimed at achieving a more comprehensive and balanced understanding of SE. They argue that SE is a multifaceted construct encompassing students’ emotions and behaviors. Additionally, SE is both a personal and context-dependent concept shaped by the socio-cultural environment (Kahu, 2013). It is also a dynamic and evolving process (Lawson and Lawson, 2013), influenced by various contextual and interpersonal factors. Therefore, in the present study, we embrace SE as a multifaceted and complex meta-construct that draws on the prominent North American model, which is an approach developed by Fredricks et al. (2004) and Fredricks (2015). This model conceptualizes SE as consisting of three interrelated first-order dimensions: behavioral, emotional, and cognitive.

In the HE context, behavioral engagement (BE) refers to participation in classroom and university-related extracurricular activities, attention, persistence, positive behavior, adherence to university rules, and the absence of disruptive behavior (Assunção et al., 2020; Esposito et al., 2022; Marôco et al., 2016; Sharif-Nia et al., 2023). Emotional engagement (EE) focuses on the negative and positive emotions associated with the learning process, including reactions to teachers and classmates, a sense of belonging to the HE institution and its subjects, and beliefs about the value of HE (Assunção et al., 2020; Esposito et al., 2022; Fredricks, 2015; Fredricks et al., 2004; Marôco et al., 2016). Cognitive engagement (CE) is defined as self-regulated learning, the use of strategies related to the learning process, and the effort required to comprehend and master complex ideas and challenging skills, as well as the development of competencies for academic activities (Assunção et al., 2020; Marôco et al., 2016). These three dimensions can be understood as existing along a continuum with both positive and negative extremes. Consequently, SE can present itself in either positive or negative forms across behavioral, cognitive, and emotional domains (Assunção et al., 2020; Esposito et al., 2022). It is important to note that these dimensions are interrelated and coexist on an equal level rather than forming a hierarchical structure, as might otherwise be implied.

## 3 Method

We conducted quantitative data analysis based on a cross-sectional study using a USEI questionnaire in Norwegian. Ethical standards per the Norwegian National Research Ethics Committees (2016) were met, and the Norwegian Agency for Shared Services in Education and Research granted permission to use the research data and carry out the study (Ref. no. 643035).

### 3.1 Procedures

SE was measured using the USEI, which was developed by Marôco et al. (2016) and subsequently adapted to various international contexts (e.g., Assunção et al., 2020; Freiberg-Hoffmann et al., 2022; Sharif-Nia et al., 2023, 2024). The USEI conceptualizes SE as a second-order construct reflected in three first-order constructs comprising behavioral, emotional, and cognitive dimensions. Each of the three first-order factors consists of five self-report items rated on a five-point Likert scale ranging from one (*never*) to five (*always*). First, the behavioral dimension (e.g., item 3: “I usually do my homework on time”) assesses students’ participation in study-related activities and classroom tasks through items eng1 to eng5. Second, the emotional factor (e.g., item 9: “I am interested in schoolwork”) assesses students’ feelings about schoolwork and their sense of belonging to the university through items eng6 to eng10. Reverse scoring was used for item 6, which is a negatively worded question. Third, the cognitive dimension (e.g., item 15: “I try to integrate subjects from different disciplines into my general knowledge”) assesses whether students are willing to invest effort into comprehending advanced ideas and skills through items eng11 to eng15.

In addition to the USEI, the questionnaire contained socio-demographic questions to describe the sample of participants and questions about academic coping to allow predictive criterion analysis using SE as a regressor. Demographic variables assessed included gender and age. Age was collected in 10-year intervals to ensure participants’ anonymity and protect their privacy. Participants were asked to state their subject of study (STEM or social sciences), type of degree (bachelor, master’s or PhD/other) and whether they were enrolled in universities or university colleges. Gender and subject of study were used as grouping variables for invariance studies. The self-reported academic variables were whether students had ever failed a class in their degree (yes/no), students’ intention to drop out of the studies, and students’ use of medication to cope with challenges related to their studies; the latter two were rated with a five-point Likert scale from one (*never*) to five (*very frequently/always*, respectively).

Despite the strong psychometric characteristics of the existing USEI, adapting it to the Norwegian context was required. First, the USEI was translated and adapted into Norwegian, because students with a poor understanding of English might misunderstand items, leading to inaccurate responses, and the nuances of items on the original USEI might change depending on the cultural setting (Nora et al., 2018). Recommendations from the literature on ensuring accurate equivalence were followed for translating the questionnaire and adapting it to the Norwegian context (Hansén and Backström-Widjeskog, 2002). More specifically, three aspects were considered: linguistic, organizational, and contextual equivalence. Linguistic equivalence was reached using a bilingual professional translator to translate the USEI into Norwegian. The translated version was then back-translated by an independent bilingual professional. Subsequently, two authors reviewed and evaluated the equivalence between the original and back-translated versions. Achieving contextual equivalence involved understanding Norwegian HE’s historical, social, political, and cultural context, while organizational equivalence was ensured by adapting the USEI to the structure of the education system and the ethical principles guiding HE in Norway. Consequently, the section on socio-demographic questions was specifically tailored to the Norwegian context.

The final Norwegian version was pilot-tested with a group of nine randomly selected university students from different courses to identify potential issues related to the clarity and comprehensibility of the items.

The Norwegian questionnaire was conducted online. The data collection process took place in 2024 and lasted 9 months with start and end date of questionnaire distribution on February 14 and November 12, respectively. The central administrations of all major public Norwegian universities and university colleges were invited to participate in the research. Those institutions that agreed to disseminate the questionnaire distributed the online survey’s URL to their students via email or their preferred learning platform. Only completed surveys were used for the analysis. On the first page of the survey, students were informed about the purpose of the study and that participation was entirely voluntary and anonymous. Students had to actively confirm that they had read the information and gave informed consent to participate (by checking a box) to be able to proceed to the questionnaire.

### 3.2 Participants

A total of 833 Norwegian students, of whom 73.1% were female, agreed to participate in the study. Most were enrolled at universities (67.9%), with the remainder attending university colleges. The participants’ age range distribution and other study-related characteristics are summarized in Table 1. Most students were enrolled in bachelor programs, followed in descending order by integrated five-year master’s programs, two-year master’s programs, and PhD or other programs. Regarding study subjects, the sample was quite balanced between the humanities and social sciences (53.8%) and STEM subjects, including basic sciences and medicine/nursing.

**Table 1:** Participants’ characteristics—distributions of age range, degree programs, study-related need for medication and dropout intention.

Variable	Category					
Age range (%)	18–24	25–34	35–44	45–54	55–64	65–74
	49.2	29.1	12.9	7.6	1.0	0.4
Degree program (%)	Bachelor	Master (5 year)	Master (2 year)	PhD or other		
	54.9	25.0	14.1	6.0		
Need for medication (%)	Never	Rarely	Sometimes	Many times	Always	
	79.8	4.9	8.2	3.0	4.1	
Drop out intention (%)	Never	Once in a while	Sometimes	Often	Very often	
	45.1	30.0	12.4	8.2	4.3	

Most participating students stated that they never used medication to cope with challenges related to their studies. Almost half of the sample reported not having any intention to drop out of their studies, whereas almost a third answered that they had thought about dropping out once in a while (see Table 1 for details). Most participants (72.6%) had never failed any university courses in their degree programs.

### 3.3 Data analysis

Evaluation of the USEI’s psychometric properties was obtained with descriptive statistics computed with the ‘skimr’ package (Waring et al., 2022) in R. Distributional properties and measures of central tendency were examined, with a particular focus on skewness (Sk) and kurtosis (Ku) for assessment of psychometric sensitivity. Absolute values of Sk below three and Ku below seven were regarded as indicating no strong deviations from normality and thus acceptable for parametric analysis (Finney and DiStefano, 2006; Marôco, 2024).

A confirmatory factor analysis (CFA) with diagonally weighted least squares (DWLS) was performed with R’s ‘lavaan’ package (Rosseel, 2012) on the polychoric correlation matrix to evaluate evidence for factorial validity in the USEI’s three-factor model. Model fit was assessed with and considered good for the following CFA indices: standardized root mean square residuals (SRMR) and root mean square error of approximation (RMSEA) below 0.08 and Tucker-Lewis fit index (TLI) and comparative fit index (CFI) larger than 0.90 (Hu and Bentler, 1999).

Evidence of convergent and discriminant validity of the three-factor engagement structure was assessed following the theoretical framework of Fornell and Larcker (1981). Convergent validity can be evaluated using the average variance extracted (AVE), which reflects the proportion of variance captured by a factor relative to the variance attributable to measurement error. The discriminant validity may be evaluated by comparing the AVE for each factor with that factor’s shared variance with other factors. Hence, an AVE by each factor greater than 0.5 and squared correlations between two factors smaller than the individual factor’s AVE were considered suggestive of convergent and discriminant validity, respectively (Fornell and Larcker, 1981).

Measurement invariance was tested across areas of study (STEM subjects and humanities and social sciences) and gender, following Cheung and Rensvold’s (2002) criteria. Analysis of invariance was conducted at four levels: configural (same factor structure across groups), metric (equal factor loadings), scalar (equal factor loadings and intercepts), and means (scalar invariance plus equal means). The absolute ΔCFI criterion (< 0.01) was used to determine whether constraints introduced at each level did not significantly worsen model fit (Cheung and Rensvold, 2002). Strong (scalar) invariance, which entails equal factor loadings and intercepts across groups, is necessary to ensure the instrument’s validity for comparisons of group means.

Guided by theoretical assumptions about SE being a second-order factor reflecting emotional, cognitive, and behavioral engagement dimensions, a second-order factor model was evaluated through CFA according to the description above. Correlations between first-order factors were first performed to support the theory-driven claim for the second-order model.

Criterion-related validity was assessed by simultaneously regressing dropout intention, number of failed university courses, and the need for medication on the second-order SE construct. The regression analysis was performed with the ‘lavaan’ package (Rosseel, 2012).

Reliability for the three first-order factors of SE was assessed with the factors’ internal consistency, expressed with Cronbach’s *α* and McDonald’s *ω*, as implemented in the ‘semTools’ package for R (Jorgensen et al., 2022). Reliability for the second-order model was assessed by McDonald’s *ω* (*ω*_L1_ and *ω*_L2_). Values of *α* and *ω* exceeding 0.7 are considered adequate levels of factor reliability for empirical research (Gadermann et al., 2012).

## 4 Results

### 4.1 Distributional properties of items

Descriptive statistics—mean, standard deviation (SD), min, max, quartile, Sk, and Ku—and histograms for the 15 USEI items (after inverting item 6) are presented in Table 2. The overall mean response of the 15 items was 3.78, which reflects central tendencies where the majority of responses cluster in the mid-range. The standard deviations ranged from 0.514 (eng2) to 1.196 (eng4), meaning that some items exhibited more diverse responses than others. The overall standard deviation of the 15 items was 1.01. The majority of items showed skewness and kurtosis absolute values below two, with the exception of item 2. None of the items displayed skewness or kurtosis values exceeding thresholds commonly associated with severe departures from normality (three and seven, respectively), suggesting adequate psychometric sensitivity (Finney and DiStefano, 2006). The items’ approximate normality justifies the subsequent use of CFA (Marôco, 2024).

**Table 2:** Distributional properties—mean, standard deviation (SD), min, max, quartiles, skewness (Sk), kurtosis (Ku)—and histograms of the USEI’s 15 items (*n* = 833).

Item	Mean	SD	Min	p25	p50	p75	Max	Sk	Ku	Histogram
eng1	4.080	0.754	1	4	4	5	5	−0.922	1.633	▁▁▂▇▃
eng2	4.747	0.514	1	5	5	5	5	−2.184	5.812	▁▁▁▂▇
eng3	4.255	0.885	1	4	4	5	5	−1.172	0.985	▁▁▂▆▇
eng4	3.114	1.196	1	2	3	4	5	−0.094	−0.892	▃▆▇▇▃
eng5	4.325	0.777	1	4	4	5	5	−1.233	1.858	▁▁▂▇▇
eng6r	3.300	0.951	1	3	3	4	5	−0.193	−0.179	▁▃▇▆▂
eng7	3.807	0.753	1	3	4	4	5	−0.577	0.662	▁▁▃▇▂
eng8	3.736	1.012	1	3	4	4	5	−0.776	0.227	▁▂▃▇▃
eng9	3.893	0.781	1	3	4	4	5	−0.583	0.599	▁▁▃▇▃
eng10	3.307	0.979	1	3	3	4	5	−0.414	−0.210	▁▃▇▇▂
eng11	3.149	1.058	1	2	3	4	5	−0.232	−0.504	▂▅▇▇▂
eng12	3.151	0.969	1	3	3	4	5	0.019	−0.188	▁▃▇▅▂
eng13	4.185	0.808	1	4	4	5	5	−0.907	0.709	▁▁▂▇▇
eng14	3.755	0.885	1	3	4	4	5	−0.458	0.075	▁▁▆▇▃
eng15	3.840	0.896	1	3	4	4	5	−0.524	0.090	▁▁▅▇▅

### 4.2 Evidence of validity related to the internal structure

The proposed three-factor model fitted the data satisfactory; see Table 3, which lists the standardized factor loadings, standard errors, z-values, and significance levels for the USEI indicators. All items demonstrated significant factor loadings (*p* < 0.001), indicating that the latent factors are strongly connected to their respective indicators. The items eng3 and eng6 (the latter was the only reversed item) had poor (<0.4) factor loadings, and eng2 had low (<0.5) factor loading, although above the minimum criterion (0.32) acceptable for factor loadings (Tabachnick and Fidell, 2013). All the remaining items had good loadings (>0.5) in each of the three dimensions, suggesting good indicator validity across the model. The intra-factor correlations were 0.73 (BE vs. EE), 0.57 (EE vs. CE), and 0.65 (BE vs. CE) between the latent behavioral-emotional, emotional-cognitive, and cognitive-behavioral engagement, respectively. All inter- and intra-factor loadings reached statistical significance at the *p* < 0.001 level.

**Table 3:** Confirmatory factor analysis, showing standardized factor loadings (*β*), standard errors (SEs), z-statistics, and *p*-values (*** indicates significance at the *p* < 0.001 level), for the three dimensions of the student engagement model.

Factor	Item	*β*	SE	Z	*p*
BE	eng1	0.616	0.066	11.912	***
BE	eng2	0.428	0.064	7.385	***
BE	eng3	0.372	0.048	8.292	***
BE	eng4	0.645	0.066	12.709	***
BE	eng5	0.615	0.069	11.355	***
EE	eng6r	0.385	0.044	9.458	***
EE	eng7	0.838	0.109	14.070	***
EE	eng8	0.637	0.052	15.808	***
EE	eng9	0.848	0.097	16.446	***
EE	eng10	0.727	0.061	17.296	***
CE	eng11	0.567	0.051	13.429	***
CE	eng12	0.617	0.055	14.132	***
CE	eng13	0.642	0.066	12.633	***
CE	eng14	0.793	0.084	15.443	***
CE	eng15	0.728	0.066	16.119	***

EE, emotional engagement; BE, behavioral engagement; CE, cognitive engagement.

The three-factor model of SE demonstrated evidence of satisfactory factorial validity. As anticipated given the sample size, the chi-square statistic (χ^2^ = 436.286, df = 87) was significant. Fit indices indicated a good model fit, with the CFI of 0.951 and TLI of 0.941 exceeding the conventional threshold of 0.90. Additionally, SRMR = 0.053 and RMSEA = 0.069 (90% CI [0.063, 0.076]) were below the recommended cutoff of 0.08, suggesting that the model provided a close approximation of the item correlations. Overall, the three-factor model of SE demonstrated a good fit to the observed data, supporting evidence of validity regarding the internal structure of the instrument.

### 4.3 Convergent related validity

Convergent related validity was observed for emotional and cognitive dimensions of SE with AVEs above 0.5 for the emotional (0.581) and slightly below 0.5 for the cognitive (0.480) dimensions, but not for the behavioral (0.317) dimension. The inter-dimensional correlations were greater than the square root of the AVE for BE and CE, as shown in Table 4, suggesting no discriminant validity, but there was discriminant validity for the emotional versus cognitive dimensions.

**Table 4:** Fornell-Larcker matrix with the square root of the average variance extracted along the main diagonal and inter dimensional correlations off-diagonal.

Factor	BE	EE	CE
BE	0.563		
EE	0.726	0.762	
CE	0.646	0.573	0.693

### 4.4 Measurement invariance

Invariance analyses for area of studies (social sciences and humanities vs. STEM subjects) and gender were conducted by testing configural, metric, scalar, and factors’ means invariance, based on ΔCFI and ΔRMSEA cutoffs (see Table 5). Strong measurement invariance of the tri-factorial SE model was displayed for gender (ΔCFI = −0.002; ΔRMSEA = −0.002) and the area of studies (ΔCFI = −0.002; ΔRMSEA = −0.002). Overall, these results support configural, metric and scalar invariance, with means invariance also retained.

**Table 5:** Gender and area of study (social sciences vs. STEM) analysis of invariance for the USEI.

Model	df	*χ* ^2^	Δ*χ*^2^	Δdf	*p*	CFI	RMSEA	ΔCFI	ΔRMSEA
Gender									
Config	174	528.460				0.887	0.070	0.000	0.000
Metric	188	548.933	16.888	14	0.262	0.885	0.068	−0.002	−0.002
Scalar	199	577.152	26.378	11	0.006	0.880	0.068	−0.005	0.000
Means	203	579.558	3.419	4	0.490	0.880	0.067	0.001	−0.001
Study area									
Config	174	532.593				0.886	0.070	0.000	0.000
Metric	188	553.696	19.328	14	0.153	0.884	0.068	−0.002	−0.002
Scalar	199	588.163	31.447	11	0.001	0.877	0.069	−0.007	0.000
Means	203	604.122	21.298	4	0.000	0.873	0.069	−0.004	0.000

### 4.5 Engagement as a second-order factor

**Figure 1:**
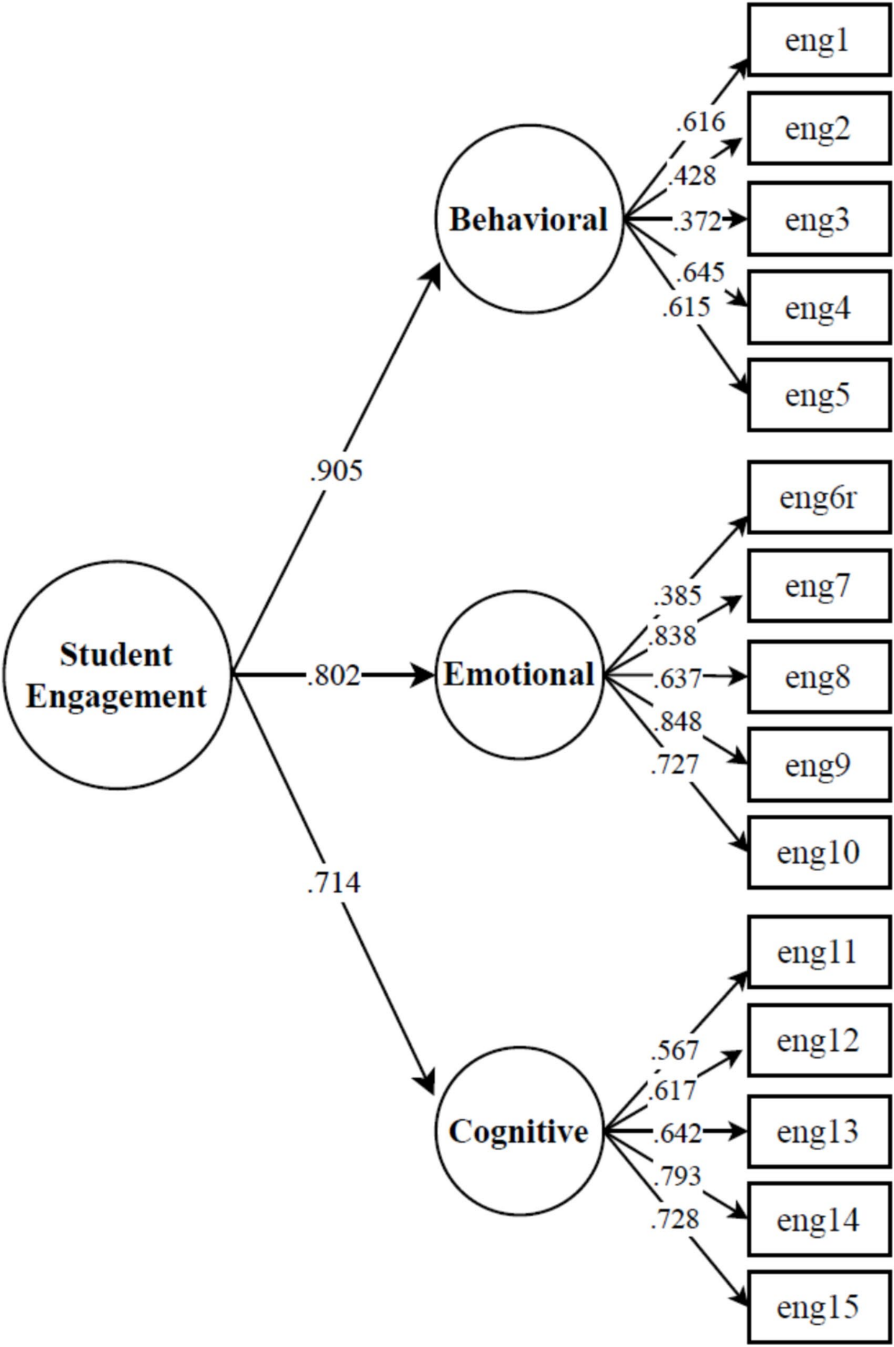
Confirmatory factor analysis of the USEI as a second-order model, showing standardized factor loadings (*β*). All indicators demonstrate significant factor loadings (*p* < 0.001).

In the presented instrument, SE is theorized as a second-order factor grounded in the three behavioral, emotional, and cognitive engagement first-order factors. There is empirical evidence for the second-order construct from the lack of discriminant validity, as shown in the previous section, and moderate to strong inter-factor correlations. Therefore, we tested the second-order factor model presented in Figure 1. Since the three-factor model indicated strong measurement invariance between the groups, the second-order model was examined on the basis of the entire sample. In Norway’s HE context, SE is primarily reflected in BE (*β* = 0.905, *p* < 0.001), while emotional (*β* = 0.802, *p* < 0.001) and cognitive (*β* = 0.714, *p* < 0.001) engagement also make strong contributions. The second-order factor model of engagement showed satisfactory overall scaled goodness of fit to the items’ variance–covariance data (*χ*^2^ = 436.29, df = 87, CFI = 0.951, TLI = 0.941, RMSEA = 0.069 [0.063, 0.076], SRMR = 0.053).

### 4.6 Criterion-related validity

The second-order SE construct showed significant negative predictive criterion validity with students’ dropout intentions (*β* = −0.510, *p* < 0.001), failed university courses (*β* = −0.304, *p* < 0.001), and use of medication to cope with study-related challenges (*β* = −0.183, *p* < 0.001).

### 4.7 Internal consistency: reliability

Table 6 presents the estimates of ordinal Cronbach’s *α* (*α*ₒᵣ_d_, based on the polychoric correlation matrix) and McDonald’s *ω* for the three first-order latent factors—behavioral, emotional, and cognitive engagement—along with the *ω* estimates for the second-order model. BE showed *α* and *ω* values below the recommended threshold of 0.70, indicating limited internal consistency. By contrast, the other first-order dimensions and the overall second-order SE construct displayed alpha and omega coefficients above 0.70, supporting acceptable internal consistency.

**Table 6:** Internal consistency and reliability of the USEI.

Construct	Reliability	BE	EE	CE	Engagement
First-order	*α*_ord_	0.662	0.803	0.794	
	*ω*	0.612	0.767	0.761	
	AVE	0.317	0.580	0.480	
Second-order	*ω*_L1_				0.744
	*ω*_L1_				0.871

## 5 Discussion

This article comprises the Norwegian validation of the USEI, an inventory designed by Marôco et al. (2016) to assess SE, which has been validated and proven reliable in countries all over the world (Assunção et al., 2020) but had not yet been tested in Norway.

The inventory showed good psychometric sensitivity as the items approximated the normal distribution. Regarding construct related validity, all 15 items exhibited adequate factor loadings that reached statistical significance (*p* < 0.001), demonstrating good indicator validity across the model. Evidence of factorial-related validity was found for both the first- and second-order models, as supported by the strong fit indices obtained for each model. Reliability analysis revealed acceptable internal consistency for SE as a second-order factor and for the first-order dimensions emotional and cognitive engagement, with Cronbach’s *α* and McDonald’s *ω* values exceeding the 0.7 threshold (Gadermann et al., 2012). However, this criterion was not met for BE, which was also the case for the Italian validity study of the USEI, which obtained a satisfactory overall goodness of fit (Esposito et al., 2022).

Despite evidence of acceptable factorial related validity, the present study has certain weaknesses regarding convergent validity. The AVE results indicate convergent validity for the emotional and cognitive dimensions of SE but not for the behavioral dimension. By contrast, discriminant validity was observed solely between the emotional and cognitive dimensions. Considering the suboptimal internal consistency of the behavioral dimension, these results suggest no strong correlation between the three first-order dimensions of SE and they may measure different aspects of the engagement construct in the Norwegian context. Previous studies also reported similar observations and found the discriminant and convergent validities for behavioral and emotional dimensions to be less than acceptable (Esposito et al., 2022; Sinval et al., 2021). However, other studies reported satisfactory discriminant and convergent validity across all dimensions (Assunção et al., 2020). The weaknesses of the results could be caused by the low factorial loadings of some items, such as items 2, 3 and 6, which could represent suboptimal indicators of the factors they are intended to measure: namely, behavioral and emotional engagement. It can be inferred that item 2 (“I follow the school’s rules”) and item 3 (“I usually do my homework on time”) do not contribute effectively to the BE factor among Norwegian students. For instance, item 2 might be interpreted differently by those students, with some viewing it as adhering to institutional rules like paying university semester fees and others interpreting it as following conventional social norms such as respecting educators and classmates. However, cultural and contextual factors may also influence these results. Norwegian students are generally older than their peers in other European countries (Haugen et al., 2024) and often combine studies with paid employment (Hauschildt et al., 2024), reflecting a high degree of independence. This pattern reflects deliberate policies that promote a flexible HE system in Norway.

Regarding item 3, students from various fields of study (e.g., health sciences, mathematics, and education) may have different approaches to assignments, with some not considering them as homework. Yet these results also align with previous findings showing that Norwegian students spend less time on academic studies than their counterparts in other Scandinavian countries (Haugen et al., 2024). Consequently, our findings reveal the need to rephrase items 2 and 3 for clearer presentation in future studies.

One possible explanation for the low factorial loading of Item 6 (“I do not feel very accomplished at this school”) is that it is the only reversed item, which could have caused issues in how participants responded. This finding aligns with other studies that report lower reliability for item 6 than for the other USEI items (Assunção et al., 2020; Sharif-Nia et al., 2023; Sinval et al., 2021). The negative impact of item reversion on the scale’s psychometric properties has been documented (Suárez Álvarez et al., 2018; Zhang et al., 2016), suggesting that, in future studies, item 6 should be presented in a manner consistent with the other items.

Invariance analyses were conducted across areas of study (social sciences and humanities vs. STEM subjects) and gender, due to different perspectives between male and female students regarding academic regulations and coursework (Vincent-Lancrin, 2008). The strong measurement invariance of the tri-factorial SE model observed for gender and areas of study confirmed the instrument’s robustness for group comparisons. The configural and mean invariance demonstrated that the same factor structure is applicable and that the instrument can reliably compare mean levels of SE for both gender and area of study. Metric and scalar invariance confirmed equivalence in factor loadings and intercepts. These findings indicate that fairness and accuracy in assessment across groups have been achieved (Cheung and Rensvold, 2002); they also align with Portuguese data (Marôco et al., 2016).

In the present study, the novelty lies in examining the relationship between student dropout rates and SE. Regarding criterion-related validity, its results indicate that the second-order SE construct negatively predicts students’ dropout intentions, failed university courses, and medication use to cope with study-related challenges, in descending order of importance. Given that 25% of Norwegian students in HE drop out during their studies (Andresen and Lervåg, 2022), it is essential to implement interventions aimed at preventing student dropout by reducing risk factors and fostering SE through supportive and protective mechanisms (Esposito et al., 2022; Christenson and Reschly, 2010; Marôco et al., 2016, 2020). Educators may seek to empower students as knowledgeable agents by fostering their learning and cognitive growth, thus creating safe pedagogical environments. Strong academic and emotional support can improve students’ academic and social outcomes. This support can also boost their feelings of competence, mastery, acceptance, and engagement (Amdal and Hanssen, 2025; Hernández-Torrano et al., 2020; Zhoc et al., 2019). These findings are important as they show that SE is a preventive factor against dropout, failed courses, and medication use.

The USEI provides HE educators with a valuable tool to measure SE and identify risk factors. It can serve as a foundation for discussions on SE and how educational programs can be structured to strengthen SE. Furthermore, it opens opportunities for intervention research, a field that is currently underrepresented in studies of higher education in Norway (Munthe, 2020). Such research designs could generate important insights into what institutions can do to support engagement. For example, how might personalized study plans, mentoring, assessment methods, and targeted interventions for motivation and stress management influence SE? At the policy level, aggregated USEI data can inform curriculum development, guide resource allocation, and support the creation of student well-being initiatives.

### 5.1 Strengths and limitations of the study

A key strength of this study lies in its rigorous methodological approach to the validation of the USEI in the Norwegian context. The use of a large and diverse sample of 833 university and university college students from various disciplines ensured broad representativeness. The instrument demonstrated strong factorial validity, acceptable internal consistency for most dimensions, and evidence of measurement invariance across gender and fields of study, supporting its applicability in diverse educational settings. Furthermore, the predictive validity of the USEI was demonstrated by its negative association with students’ dropout intentions, failed courses, and use of medication to cope with academic challenges—highlighting its practical relevance for policies and interventions.

Although the present study offers valuable evidence regarding the internal structure of the USEI in a Norwegian sample, several psychometric limitations must be acknowledged.

This study is based on a voluntary online survey and is therefore subject to potential self-selection effects. Consequently, the sample may be skewed toward more engaged students, while the perspectives of less engaged students may be underrepresented. Such limitations are common in online research and do not necessarily imply substantial bias in the estimation of associations between variables (Groves and Peytcheva, 2008) as is the case of the present study. Nevertheless, caution is warranted when considering the generalizability of the findings. Future research should employ mixed-mode data collection strategies or institutional sampling approaches to enhance representativeness and to further assess the robustness of the results across diverse student populations.

A few BE items (e.g., items 2 and 3), which may be culturally misaligned or ambiguously worded in the Norwegian context, as well as the reversed item (item 6) demonstrated suboptimal factor loadings, reflecting potential response bias or confusion—an issue consistent with findings in international studies. These challenges suggest the need for refinement of specific items in future adaptations. Nevertheless, overall model evaluation was based on a comprehensive set of recommended fit indices, including CFI, TLI, RMSEA (with confidence intervals), and SRMR, which align with contemporary guidelines for acceptable to good model fit (Hu and Bentler, 1999; Kline, 2016).

Moreover, we recognize that validation is an ongoing, cumulative process. A single cross-sectional study can provide only partial evidence, typically related to internal structure and associations with a restricted set of external variables. Comprehensive validation requires multiple sources of evidence, such as response-process analyses, test–retest reliability, cross-sample and cross-cultural replication, and potentially item response theory (IRT) modelling, as outlined in the Standards for Educational and Psychological Testing (American Educational Research Association and American Psychological Association, and National Council on Measurement in Education, 2014). We recommend that future studies should have a longitudinal approach to track temporal SE fluctuations.

## 6 Conclusion

This study has demonstrated that the 15-item USEI can be used as a valid tool for assessing SE in the context of Norwegian HE. While its reliability requires further investigation, as one dimension appears to function more effectively than the others, the results show that the USEI supports the three-factorial structure of SE and indicate that this structure represents a second-order construct. The findings provide substantial evidence for the validity and reliability of the USEI within a Norwegian context, evaluated using a sample of 833 university and university college students. Furthermore, the evidence of measurement invariance confirms that the inventory can be reliably used across diverse student populations, facilitating cross-group comparisons and promoting equity in evaluation. The results indicated SE’s negative predictive validity regarding students’ dropout intentions, failed university courses, and medication use to cope with study-related challenges.

Considering this, a concluding summary from this article emphasizes that high dropout rates, failed university courses, and medication use among students in HE are increasing not only in Norway but also globally. Therefore, it is essential to implement strategies at the university and policy levels that assist students, enhancing their academic, personal, and professional empowerment and ultimately improving the diversity and effectiveness of HE systems. The USEI can be beneficial since it is a free, concise tool that is easy to administer for both small groups and large-scale assessments. It can be deployed to assess interventions aimed at university students and to conduct additional research in academic success, retention, and motivation among students.

## Data Availability

The raw data supporting the conclusions of this article will be made available by the authors, without undue reservation.
